# Supplementary material for: Pyruvate Dehydrogenase Kinase 1 inhibition mediated oxidative phosphorylation enhancement in cartilage promotes osteoarthritis progression
Source: BMC Musculoskelet Disord. 2023 Jul 20;24:597. doi: 10.1186/s12891-023-06585-6 (PMC10357736; doi:10.1186/s12891-023-06585-6)
Supplement: Supplementary file 1 — Additional file 1. [file 12891_2023_6585_MOESM1_ESM.zip › MF.Gsea.1653627316242/GOMF_SEROTONIN_RECEPTOR_ACTIVITY.html]

Details for gene set GOMF\_SEROTONIN\_RECEPTOR\_ACTIVITY[GSEA]

|  || Dataset | 55457\_collapsed\_to\_symbols.cls.cls #OA\_versus\_normal.cls.cls #OA\_versus\_normal\_repos |
| Phenotype | cls.cls#OA\_versus\_normal\_repos |
| Upregulated in class | normal |
| GeneSet | GOMF\_SEROTONIN\_RECEPTOR\_ACTIVITY |
| Enrichment Score (ES) | -0.44812107 |
| Normalized Enrichment Score (NES) | -1.4482965 |
| Nominal p-value | 0.05042017 |
| FDR q-value | 0.23249562 |
| FWER p-Value | 1.0 |
Table: GSEA Results Summary

  

Fig 1: Enrichment plot: GOMF\_SEROTONIN\_RECEPTOR\_ACTIVITY      
 Profile of the Running ES Score & Positions of GeneSet Members on the Rank Ordered List

  

| SYMBOL | TITLE | RANK IN GENE LIST | RANK METRIC SCORE | RUNNING ES | CORE ENRICHMENT || 1 | HTR2B | 5-hydroxytryptamine receptor 2B [Source:HGNC Symbol;Acc:HGNC:5294] | 562 | 0.636 | 0.0366 | No |
| 2 | HTR2A | 5-hydroxytryptamine receptor 2A [Source:HGNC Symbol;Acc:HGNC:5293] | 1847 | 0.379 | -0.0153 | No |
| 3 | HTR1E | 5-hydroxytryptamine receptor 1E [Source:HGNC Symbol;Acc:HGNC:5291] | 3245 | 0.226 | -0.0953 | No |
| 4 | DRD4 | dopamine receptor D4 [Source:HGNC Symbol;Acc:HGNC:3025] | 3267 | 0.224 | -0.0686 | No |
| 5 | CHRM2 | cholinergic receptor muscarinic 2 [Source:HGNC Symbol;Acc:HGNC:1951] | 3473 | 0.205 | -0.0587 | No |
| 6 | HTR2C | 5-hydroxytryptamine receptor 2C [Source:HGNC Symbol;Acc:HGNC:5295] | 3873 | 0.167 | -0.0686 | No |
| 7 | OR10H1 | olfactory receptor family 10 subfamily H member 1 [Source:HGNC Symbol;Acc:HGNC:8172] | 4167 | 0.141 | -0.0735 | No |
| 8 | CHRM4 | cholinergic receptor muscarinic 4 [Source:HGNC Symbol;Acc:HGNC:1953] | 5312 | 0.046 | -0.1565 | No |
| 9 | HTR1D | 5-hydroxytryptamine receptor 1D [Source:HGNC Symbol;Acc:HGNC:5289] | 5888 | 0.003 | -0.2008 | No |
| 10 | OR10H3 | olfactory receptor family 10 subfamily H member 3 [Source:HGNC Symbol;Acc:HGNC:8174] | 6287 | -0.026 | -0.2284 | No |
| 11 | CHRM3 | cholinergic receptor muscarinic 3 [Source:HGNC Symbol;Acc:HGNC:1952] | 8034 | -0.158 | -0.3441 | No |
| 12 | HRH1 | histamine receptor H1 [Source:HGNC Symbol;Acc:HGNC:5182] | 8146 | -0.166 | -0.3317 | No |
| 13 | HRH4 | histamine receptor H4 [Source:HGNC Symbol;Acc:HGNC:17383] | 8852 | -0.221 | -0.3586 | No |
| 14 | OR10H2 | olfactory receptor family 10 subfamily H member 2 [Source:HGNC Symbol;Acc:HGNC:8173] | 10006 | -0.322 | -0.4075 | Yes |
| 15 | HTR1A | 5-hydroxytryptamine receptor 1A [Source:HGNC Symbol;Acc:HGNC:5286] | 10008 | -0.322 | -0.3670 | Yes |
| 16 | HTR7 | 5-hydroxytryptamine receptor 7 [Source:HGNC Symbol;Acc:HGNC:5302] | 10399 | -0.360 | -0.3518 | Yes |
| 17 | CHRM5 | cholinergic receptor muscarinic 5 [Source:HGNC Symbol;Acc:HGNC:1954] | 10725 | -0.396 | -0.3271 | Yes |
| 18 | HTR1B | 5-hydroxytryptamine receptor 1B [Source:HGNC Symbol;Acc:HGNC:5287] | 10971 | -0.427 | -0.2923 | Yes |
| 19 | HTR6 | 5-hydroxytryptamine receptor 6 [Source:HGNC Symbol;Acc:HGNC:5301] | 11034 | -0.435 | -0.2423 | Yes |
| 20 | HTR1F | 5-hydroxytryptamine receptor 1F [Source:HGNC Symbol;Acc:HGNC:5292] | 11290 | -0.470 | -0.2029 | Yes |
| 21 | HRH3 | histamine receptor H3 [Source:HGNC Symbol;Acc:HGNC:5184] | 11827 | -0.554 | -0.1746 | Yes |
| 22 | HRH2 | histamine receptor H2 [Source:HGNC Symbol;Acc:HGNC:5183] | 11879 | -0.567 | -0.1071 | Yes |
| 23 | HTR4 | 5-hydroxytryptamine receptor 4 [Source:HGNC Symbol;Acc:HGNC:5299] | 12308 | -0.680 | -0.0546 | Yes |
| 24 | HTR5A | 5-hydroxytryptamine receptor 5A [Source:HGNC Symbol;Acc:HGNC:5300] | 12599 | -0.797 | 0.0234 | Yes |
Table: GSEA details [plain text format]

  

Fig 2: GOMF\_SEROTONIN\_RECEPTOR\_ACTIVITY      
 Blue-Pink O' Gram in the Space of the Analyzed GeneSet

  

Fig 3: GOMF\_SEROTONIN\_RECEPTOR\_ACTIVITY: Random ES distribution      
 Gene set null distribution of ES for **GOMF\_SEROTONIN\_RECEPTOR\_ACTIVITY**
